# Supplementary material for: Adjuvant Transarterial Chemoembolization Following Curative-Intent Hepatectomy Versus Hepatectomy Alone for Hepatocellular Carcinoma: A Systematic Review and Meta-Analysis of Randomized Controlled Trials
Source: Cancers (Basel). 2021 Jun 15;13(12):2984. doi: 10.3390/cancers13122984 (PMC8232114; doi:10.3390/cancers13122984)
Supplement: Supplementary file 1 [file cancers-13-02984-s001.zip › cancers-1244688-supplementary.pdf]

Table S1. PRISMA 2009 Checklist

Section/topic	#	Checklist item	Reported on page #
**TITLE**			
Title	1	Identify the report as a systematic review, meta-analysis, or both.	Manuscript Page 1
**ABSTRACT**			
Structured summary	2	Provide a structured summary including, as applicable: background; objectives; data sources; study eligibility criteria, participants, and interventions; study appraisal and synthesis methods; results; limitations; conclusions and implications of key findings; systematic review registration number.	Manuscript Page 1
**INTRODUCTION**			
Rationale	3	Describe the rationale for the review in the context of what is already known.	Manuscript Page 2
Objectives	4	Provide an explicit statement of questions being addressed with reference to participants, interventions, comparisons, outcomes, and study design (PICOS).	Manuscript Page 2
**METHODS**			
Protocol and registration	5	Indicate if a review protocol exists, if and where it can be accessed (e.g., Web address), and, if available, provide registration information including registration number.	Manuscript Page 2
Eligibility criteria	6	Specify study characteristics (e.g., PICOS, length of follow-up) and report characteristics (e.g., years considered, language, publication status) used as criteria for eligibility, giving rationale.	Manuscript Pages 2-3
Information sources	7	Describe all information sources (e.g., databases with dates of coverage, contact with study authors to identify additional studies) in the search and date last searched.	Manuscript Page 3, Figure 1
Search	8	Present full electronic search strategy for at least one database, including any limits used, such that it could be repeated.	Manuscript Page 3, Figure 1
Study selection	9	State the process for selecting studies (i.e., screening, eligibility, included in systematic review, and, if applicable, included in the meta-analysis).	Manuscript Page 3, Figure 1
Data collection process	10	Describe method of data extraction from reports (e.g., piloted forms, independently, in duplicate) and any processes for obtaining and confirming data from investigators.	Manuscript Page 3
Data items	11	List and define all variables for which data were sought (e.g., PICOS, funding sources) and any assumptions and simplifications made.	Manuscript Page 3, Table 1

Risk of bias in individual studies	12	Describe methods used for assessing risk of bias of individual studies (including specification of whether this was done at the study or outcome level), and how this information is to be used in any data synthesis.	Manuscript Page 3
Summary measures	13	State the principal summary measures (e.g., risk ratio, difference in means).	Manuscript Pages 3-4
Synthesis of results	14	Describe the methods of handling data and combining results of studies, if done, including measures of consistency (e.g., I^2) for each meta-analysis.	Manuscript Pages 4
Risk of bias across studies	15	Specify any assessment of risk of bias that may affect the cumulative evidence (e.g., publication bias, selective reporting within studies).	Manuscript Page 4
Additional analyses	16	Describe methods of additional analyses (e.g., sensitivity or subgroup analyses, meta-regression), if done, indicating which were pre-specified.	Manuscript Page 4
**RESULTS**			
Study selection	17	Give numbers of studies screened, assessed for eligibility, and included in the review, with reasons for exclusions at each stage, ideally with a flow diagram.	Manuscript Page 4, Figure 1
Study characteristics	18	For each study, present characteristics for which data were extracted (e.g., study size, PICOS, follow-up period) and provide the citations.	Manuscript Page 4, Table 1
Risk of bias within studies	19	Present data on risk of bias of each study and, if available, any outcome level assessment (see item 12).	Manuscript Pages 4-5, Figure 2
Results of individual studies	20	For all outcomes considered (benefits or harms), present, for each study: (a) simple summary data for each intervention group (b) effect estimates and confidence intervals, ideally with a forest plot.	Manuscript Page 5, Figures 3-4
Synthesis of results	21	Present results of each meta-analysis done, including confidence intervals and measures of consistency.	Manuscript Page 5, Figures 3-4,
Risk of bias across studies	22	Present results of any assessment of risk of bias across studies (see Item 15).	Manuscript Page 5, Supplemental Figures 1-2
Additional analysis	23	Give results of additional analyses, if done (e.g., sensitivity or subgroup analyses, meta-regression [see Item 16]).	Supplemental Figures 3-11
**DISCUSSION**			

Summary of evidence	24	Summarize the main findings including the strength of evidence for each main outcome; consider their relevance to key groups (e.g., healthcare providers, users, and policy makers).	Manuscript Pages 12-13
Limitations	25	Discuss limitations at study and outcome level (e.g., risk of bias), and at review-level (e.g., incomplete retrieval of identified research, reporting bias).	Manuscript Page 13
Conclusions	26	Provide a general interpretation of the results in the context of other evidence, and implications for future research.	Manuscript Pages 13-14
**FUNDING**			
Funding	27	Describe sources of funding for the systematic review and other support (e.g., supply of data); role of funders for the systematic review.	Manuscript Page 14

Table S2. Quality of evidence assessment using the GRADE approach.

Certainty assessment							№ of patients		Effect		Certainty	Importance
**№ of studies**	**Study design**	**Risk of bias**	**Inconsistency**	**Indirectness**	**Imprecision**	**Other considerations**	**adjuvant TACE plus curative hepatectomy**	**curative hepatectomy alone**	**Relative (95% CI)**	**Absolute (95% CI)**		
**Overall Survival (assessed with: Hazard Ratio [HR])**												
7	Randomised Controlled Trials	Serious [a]	Not serious	Not serious	Not serious	None	464 participants	479 participants	**HR 0.66** (0.52 to 0.85) [Overall Survival]	**-- per 1,000** (from -- to --)	⨁⨁⨁◯ MODERATE	
							-	-		**-- per 1,000** (from -- to --)		
**Recurrence-Free Survival (assessed with: Hazard Ratio [HR])**												
9	Randomised Controlled Trials	Serious [b]	Not serious	Not serious	Not serious	None	546 participants	566 participants	**HR 0.70** (0.56 to 0.88) [Recurrence-Free Survival]	**-- per 1,000** (from -- to --)	⨁⨁⨁◯ MODERATE	
							-	-		**-- per 1,000** (from -- to --)		

CI: Confidence interval; **HR:** Hazard Ratio

Explanations

a. The quality of evidence was downgraded by one level, as the overall risk of bias was characterized as "Some concerns" for the majority of the included studies in this outcome (5/7) and "High" in the remaining studies (2/7) using the Risk of Bias 2.0 (RoB 2.0) tool.
b. a. The quality of evidence was downgraded by one level, as the overall risk of bias was characterized as "Some concerns" for the majority of the included studies in this outcome (6/9) and "High" in the remaining studies (3/9) using the Risk of Bias 2.0 (RoB 2.0) tool.

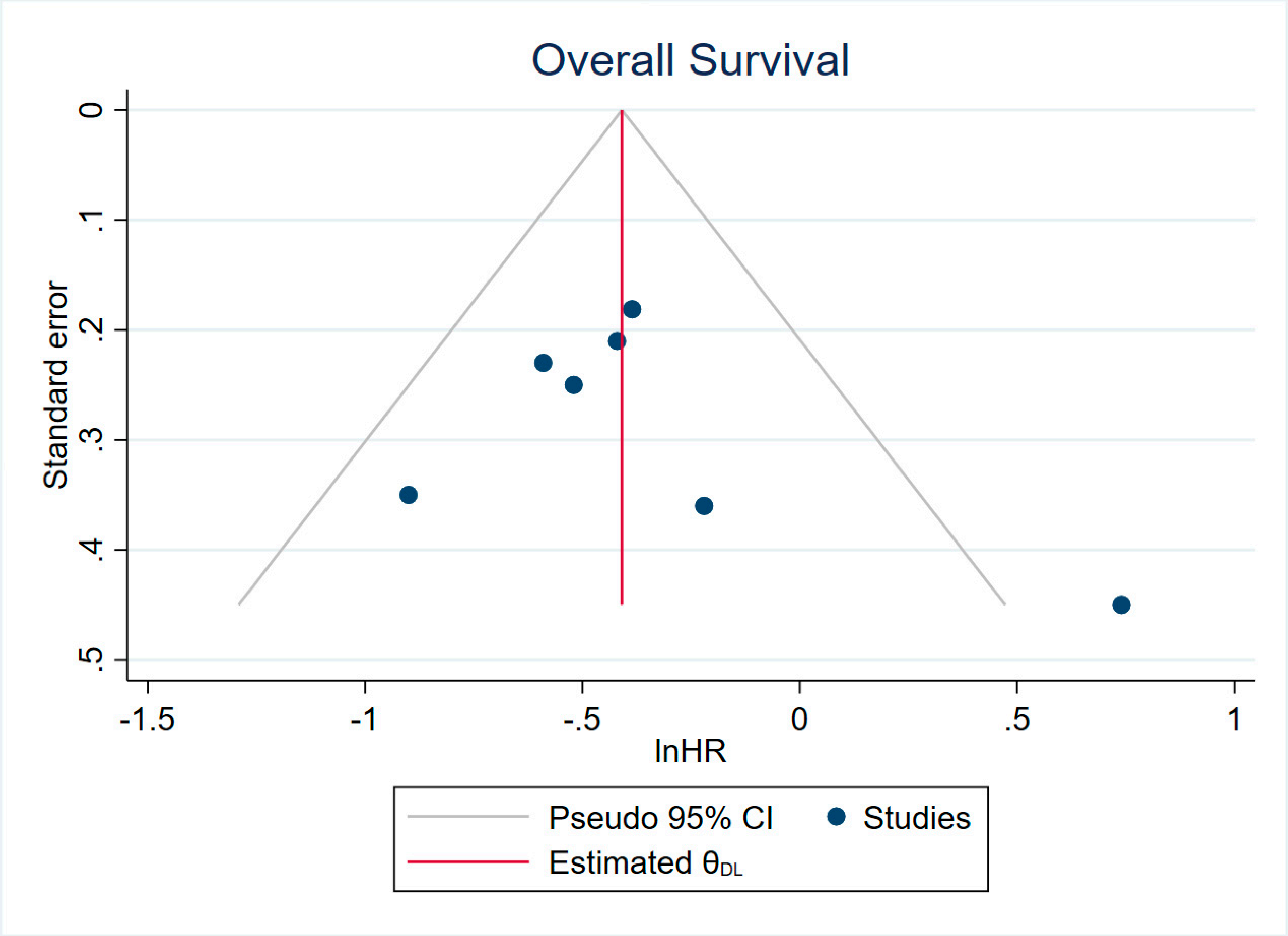

Figure S1. Funnel plot of overall survival. HR: hazard ratio; CI: confidence interval

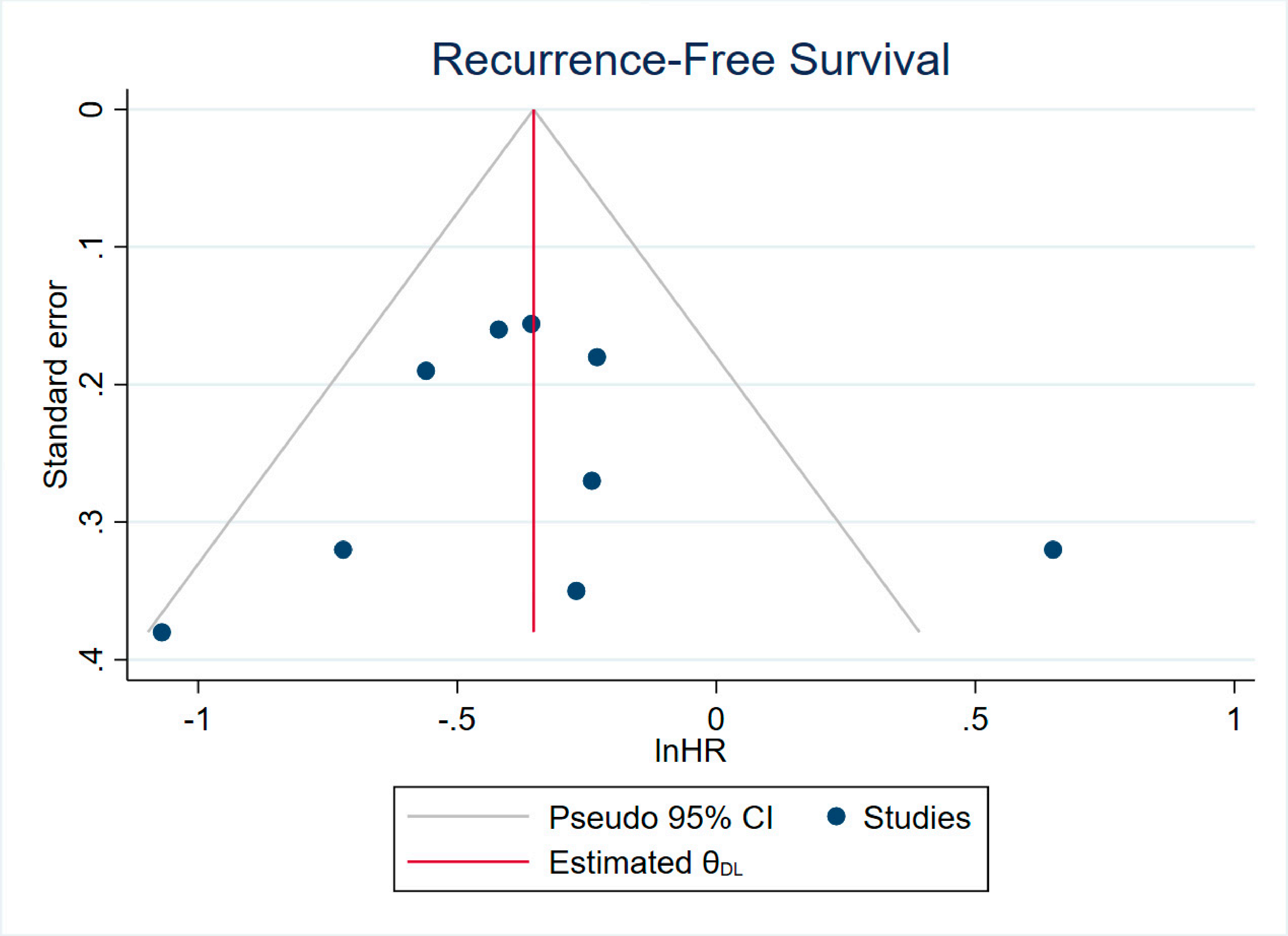

Figure S2. Funnel plot of recurrence-free survival. HR: hazard ratio; CI: confidence interval

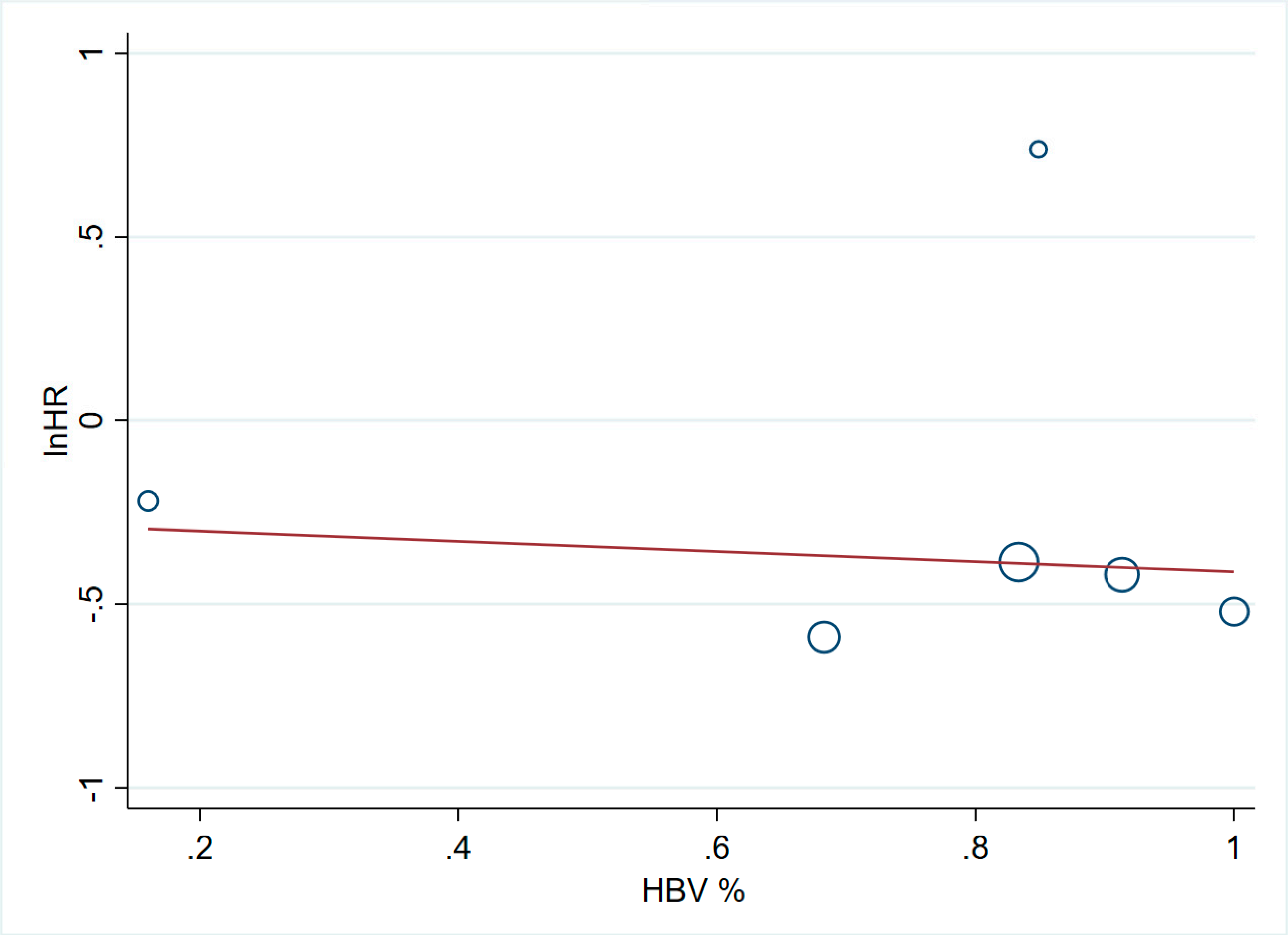

Figure S3. Meta-regression of overall survival according to the percentage of HBV-positive patients (p = 0.84). HR: hazard ratio; HBV: hepatitis B virus

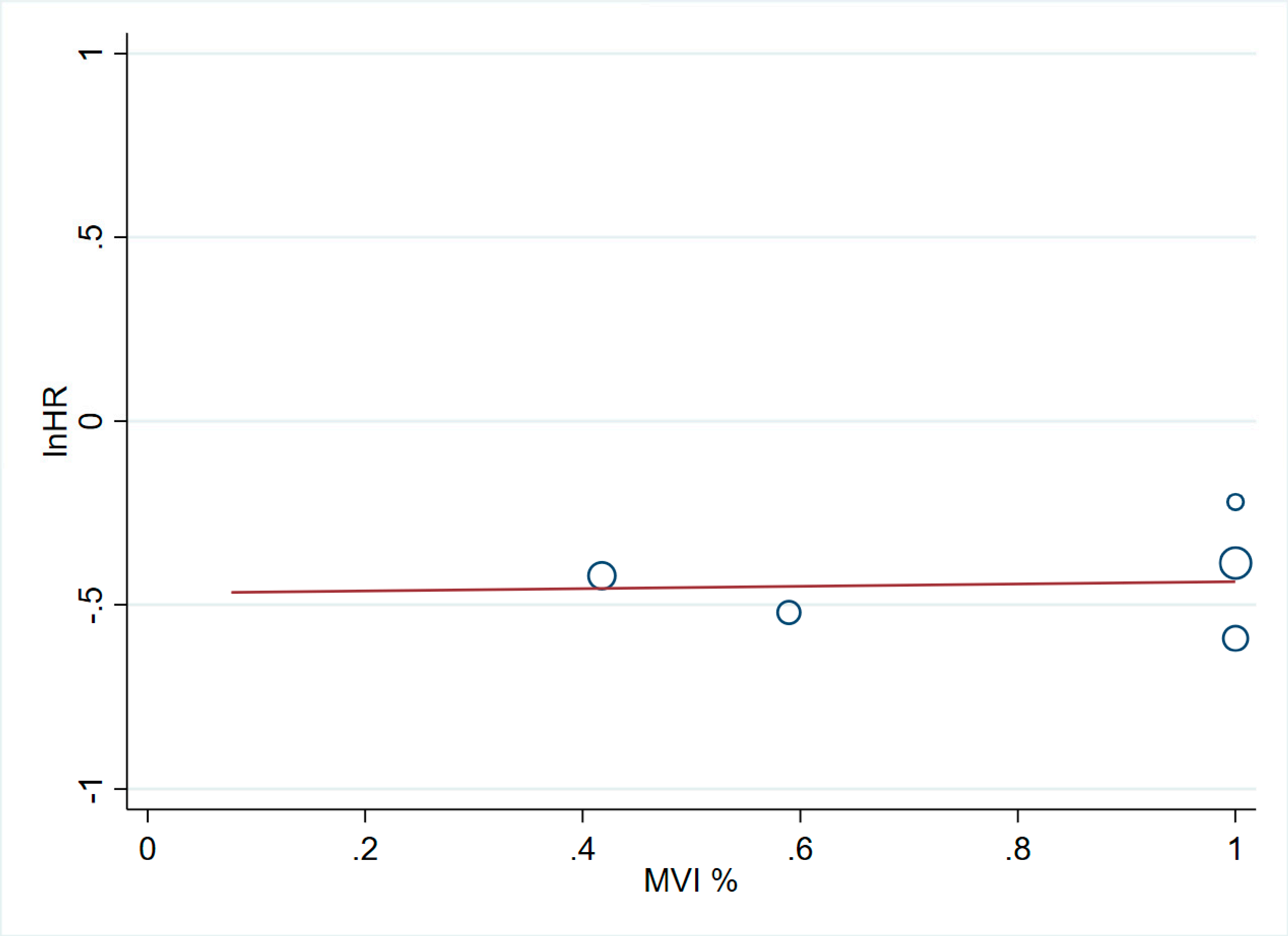

Figure S4. Meta-regression of overall survival according to the percentage of patients with microvascular invasion (p = 0.94). HR: hazard ratio; MVI: microvascular invasion

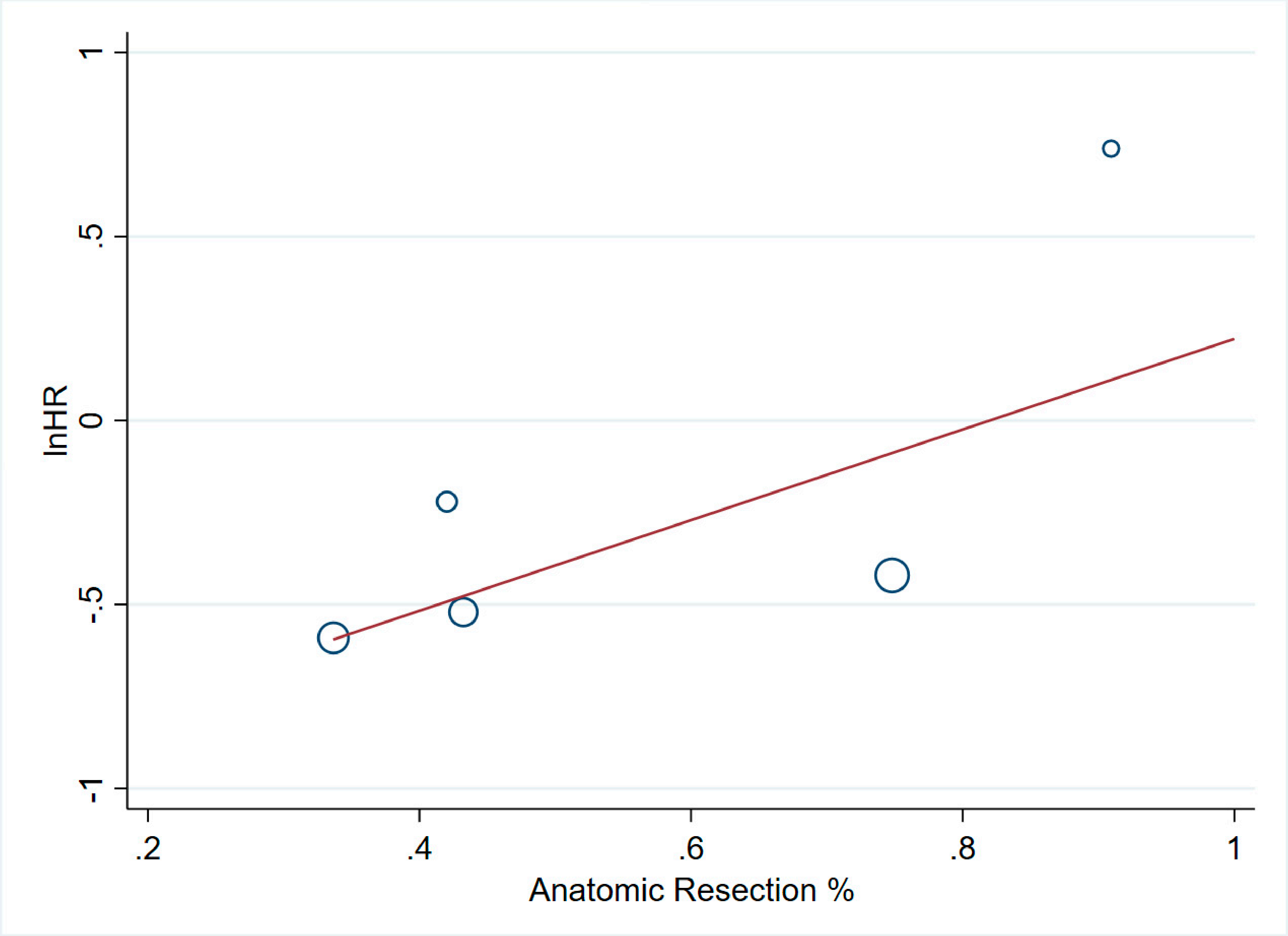

Figure S5. Meta-regression of overall survival according to the percentage of anatomic resections ($p = 0.23$). HR: hazard ratio

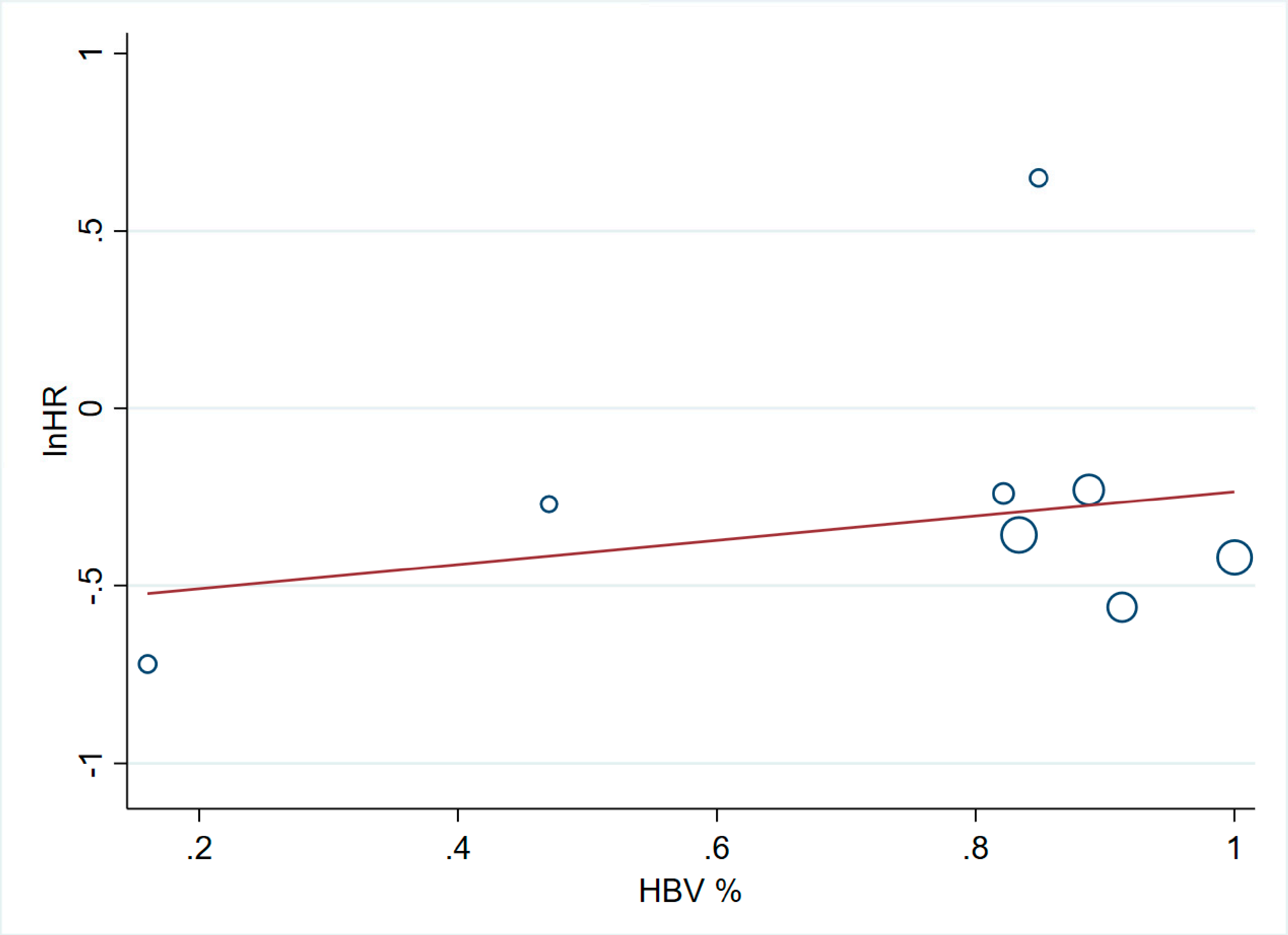

Figure S6. Meta-regression of recurrence-free survival according to the percentage of HBV-positive patients (p = 0.56). HR: hazard ratio; HBV: hepatitis B virus

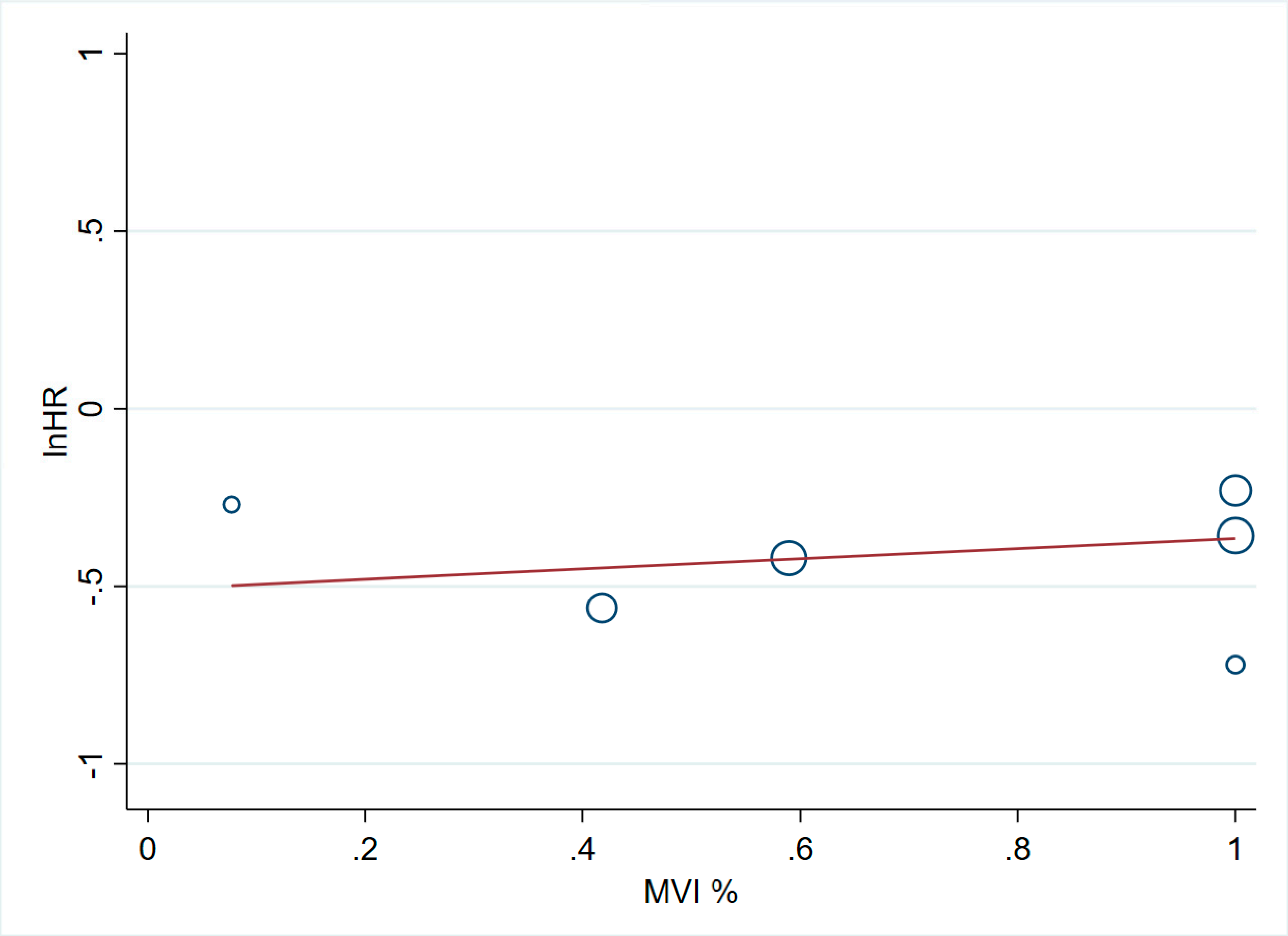

Figure S7. Meta-regression of recurrence-free survival according to the percentage of patients with microvascular invasion ($p = 0.63$). HR: hazard ratio; MVI: microvascular invasion

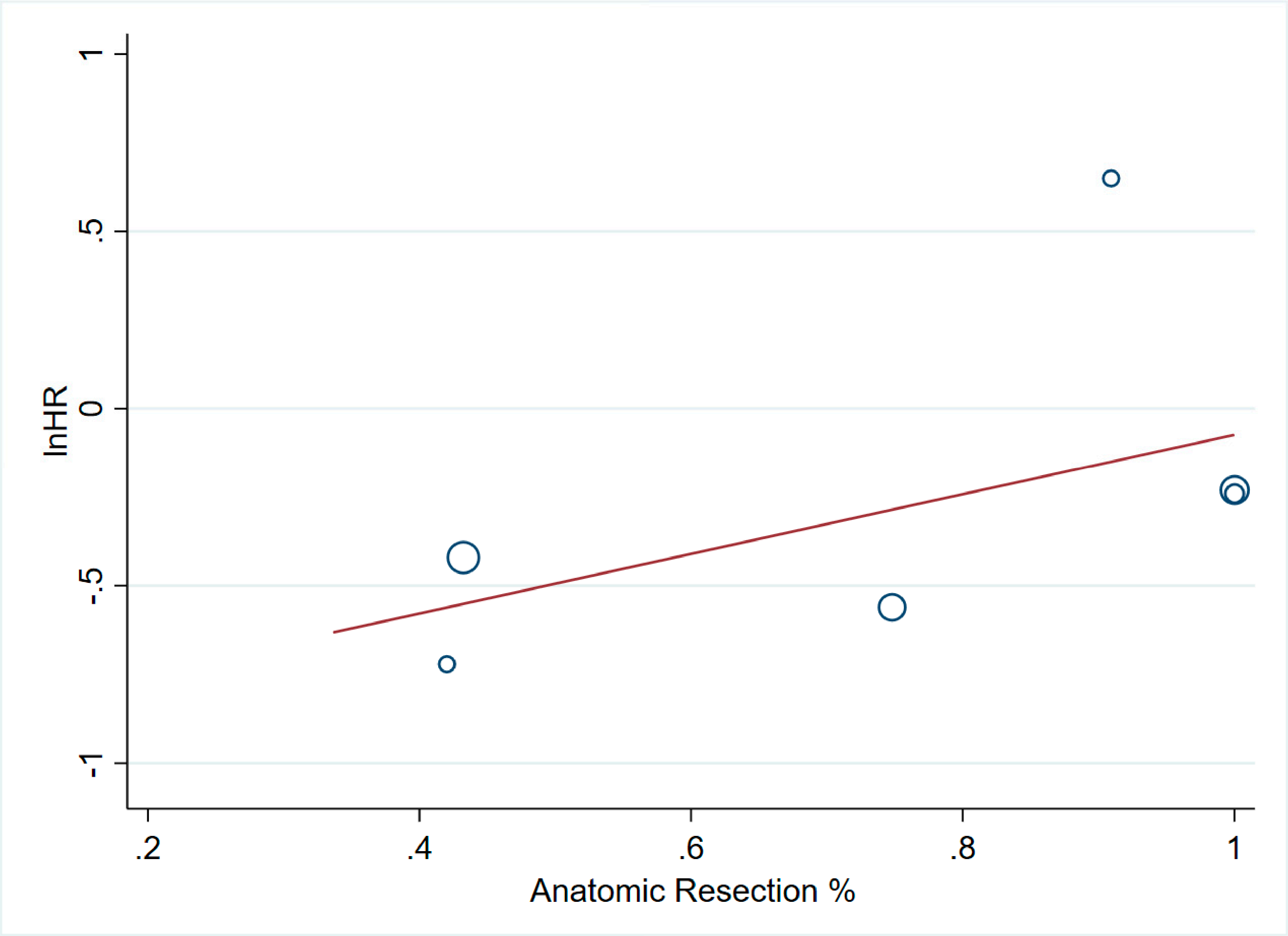

Figure S8. Meta-regression of recurrence-free survival according to the percentage of anatomic resections ($p = 0.28$). HR: hazard ratio

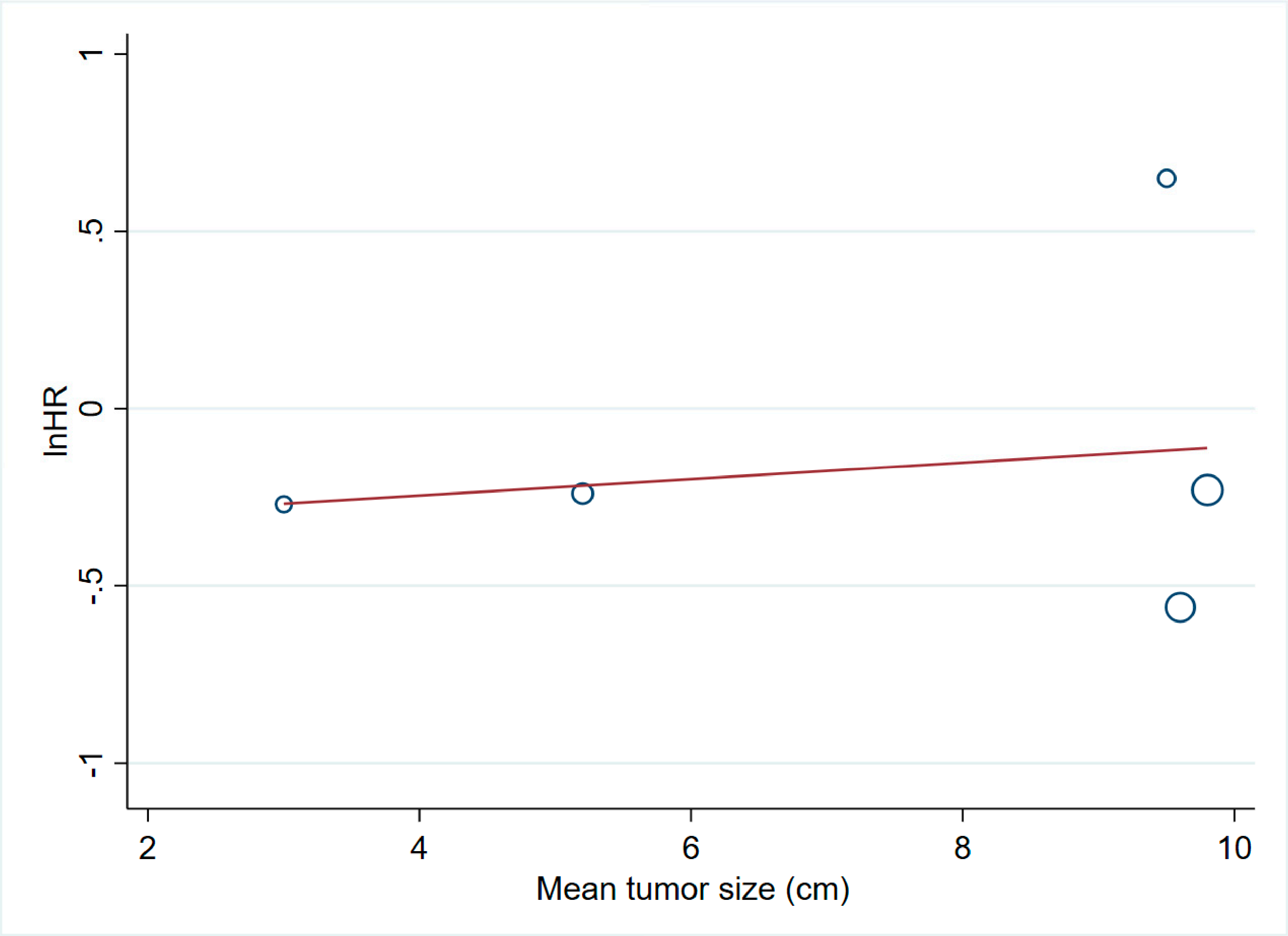

Figure S9. Meta-regression of recurrence-free survival according to mean tumor size ($p = 0.80$). HR: hazard ratio

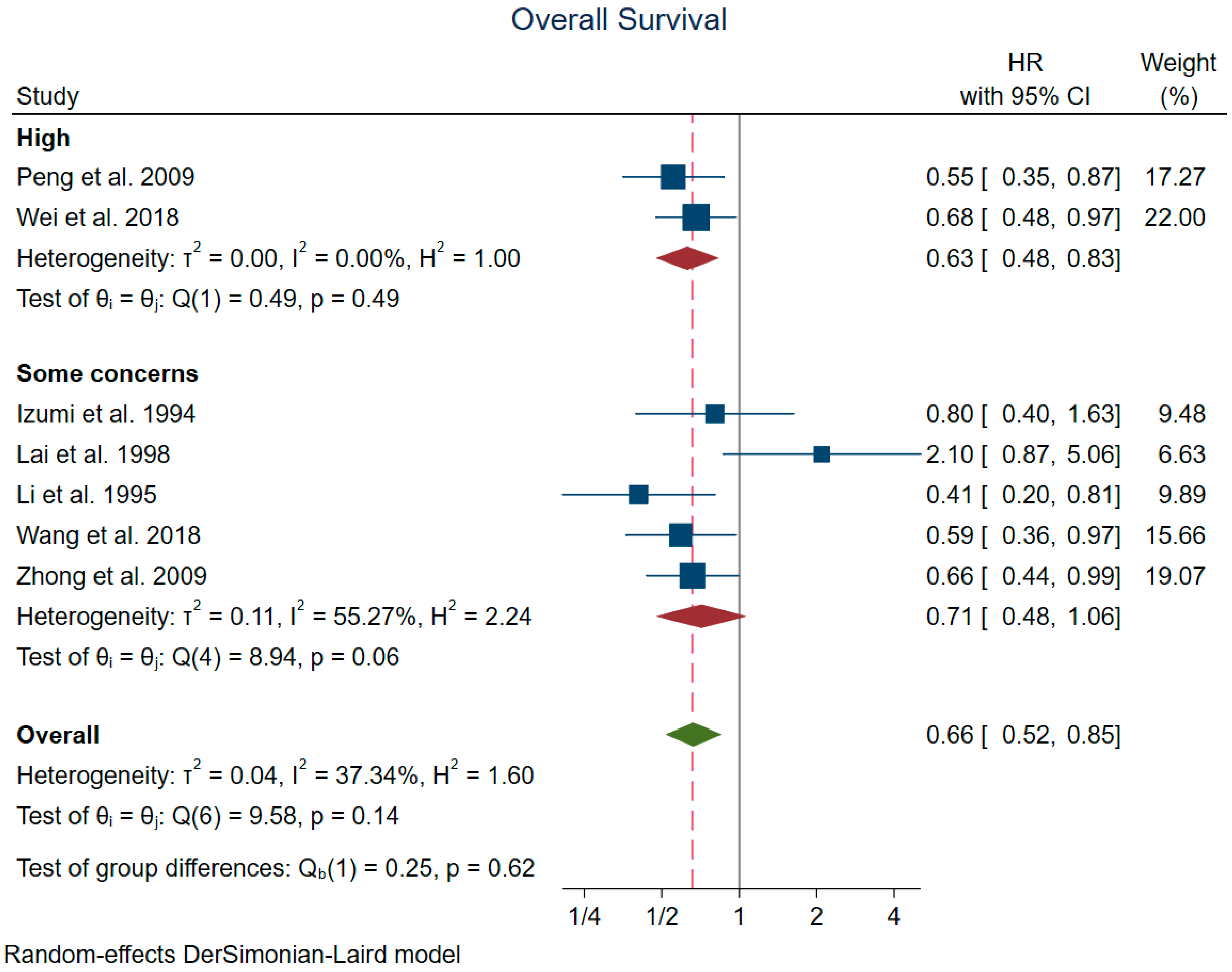

Figure S10. Subgroup analysis of overall survival according to risk of bias using the Risk of Bias 2.0 tool. HR: hazard ratio; CI: confidence interval

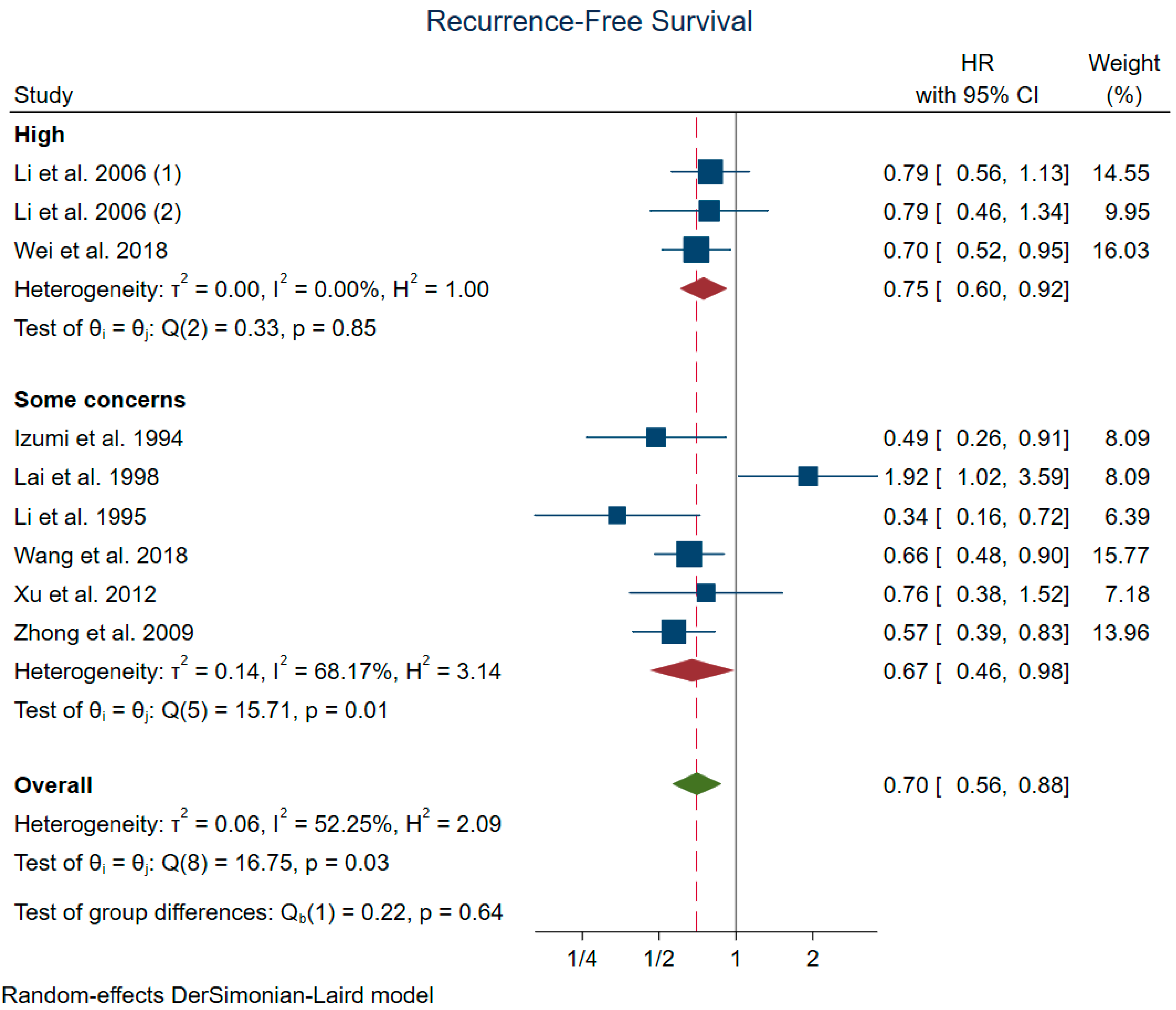

Figure S11. Subgroup analysis of recurrence-free survival according to risk of bias using the Risk of Bias 2.0 tool. HR: hazard ratio; CI: confidence interval
